# VISTA expression associated with CD8 confers a favorable immune microenvironment and better overall survival in hepatocellular carcinoma

**DOI:** 10.1186/s12885-018-4435-1

**Published:** 2018-05-02

**Authors:** Ming Zhang, Hua-Jin Pang, Wei Zhao, Yu-Fa Li, Li-Xu Yan, Zhong-Yi Dong, Xiao-Feng He

**Affiliations:** 1 Department of Interventional Radiology, Nanfang Hospital, Southern Medical University, 1838, North Guangzhou Avenue, Guangzhou, China; 2 Department of Radiation Oncology, Nanfang Hospital, Southern Medical University, 1838, North Guangzhou Avenue, Guangzhou, China; 3 Department of Pathology and Laboratory Medicine, Guangdong General Hospital and Guangdong Academy of Medical Sciences, 106 Zhongshan Er Rd, Guangzhou, China

**Keywords:** VISTA, Hepatocellular carcinoma, CD8+ tumor-infiltrating lymphocytes, Tumor microenvironment, Prognosis

## Abstract

**Background:**

Hepatocellular carcinoma (HCC) often arises in the setting of chronic inflammation with multiple inhibitory immune signals. V-domain Ig suppressor of T cell activation (VISTA) is identified as a novel negative checkpoint regulator. This study sought to determine the expression and prognostic value of VISTA in HCC and classify tumor microenvironments (TMEs) based on VISTA and CD8+ tumor-infiltrating lymphocytes (TILs).

**Methods:**

The expression of VISTA and CD8 proteins was assessed in 183 HCC tissue microarrays (TMAs) by immunohistochemistry (IHC). *VISTA* and *CD8A* mRNA extracted from 372 patients with HCC in The Cancer Genome Atlas (TCGA) database was included as a validation cohort. Associations between the VISTA, clinicopathological variables, and survival were analyzed.

**Results:**

VISTA expression was detected in 29.5% HCC tissues, among which 16.4% tissues were positive for tumor cells (TCs), and 16.9% tissues were positive for immune cells (ICs). VISTA expression was significantly associated with tissues with a high pathological grading (*p* = 0.038), without liver cirrhosis (*p* = 0.011), and with a high density of CD8 + TILs (*p* < 0.001). Kaplan-Meier curves demonstrated that patients with VISTA-positive staining in TCs (*p* = 0.037), but not in ICs, (*p* = 0.779) showed significantly prolonged overall survival (OS) than those with VISTA-negative expression. Classification of HCC TME-based VISTA and CD8 + TILs showed 4 immune subtypes: VISTA+/CD8+ (16.9%), VISTA+/CD8- (12.6%), VISTA-/CD8+ (16.4%), and VISTA-/CD8+ (54.1%). The dual positive VISTA+/CD8+ subtype showed significantly prolonged OS than other subtypes (*p* = 0.023).

**Conclusions:**

VISTA protein expression in HCC showed cell specific and displayed different prognosis. VISTA expression was significantly associated with CD8 + TILs, Dual positive VISTA+/CD8+ showed favorable TME and better OS.

**Electronic supplementary material:**

The online version of this article (10.1186/s12885-018-4435-1) contains supplementary material, which is available to authorized users.

## Background

Hepatocellular carcinoma (HCC) is the leading cause of cancer-related mortality worldwide. Radical surgery and liver transplantation are only available in those with early-stage HCC, for most patients with advanced disease, the current systemic treatment options provide limited therapeutic benefits, and hence, novel therapeutic options are needed [1, 2]. Fortunately, recent clinical trials with immune checkpoint blockade therapies have shown unprecedented treatment responses to many types of cancers. The CheckMate 040 is the first study that confirmed the safety and favorable efficacy of Nivolumab, a programmed death 1 (PD-1) inhibitor, in patients with advanced HCC [3]. These studies highlight a promising method to treat HCC based on immune checkpoint blockades.

The occurrence and development of HCC is generally correlated with inflammatory stimulation characterized by close communication between the tumor cells and the inflammatory microenvironment consisting of transformed epithelial cells, tumor-associated fibroblasts, and immunosuppressive macrophages [4]. It is of great importance to understand the role and status of immune checkpoints in HCC microenvironments and whether we can target these immune checkpoints to enhance anti-tumor effects. Previous studies have proposed that tumor microenvironments exist based on the presence or absence of tumor-infiltrating lymphocytes (TILs) and Programmed cell death 1 ligand 1 (PD-L1) expression [5, 6]. Dual positive of PD-L1 and TILs were defined as an inflamed phenotype microenvironment, which demonstrated the best response to anti-PD-1/L1 therapy [7, 8].

Recent studies have identified that V-domain Ig suppressor of T cell activation (VISTA) is a novel negative checkpoint regulator, which shared homology with PD-L1 and potently suppressed T-cell activation. VISTA is expressed predominantly on hematopoietic cells, e.g. myeloid, granulocytic and T cells [9, 10]. Its levels are heightened within the tumor microenvironment, in which its blockade can enhance antitumor immune responses in mice [11]. Interestingly, VISTA-induced T cell inactivation seemed to be nonredundantly from the PD-1/PD-L1 pathway [12, 13]. Meanwhile, VISTA levels were shown to increase after ipilimumab therapy in patients with prostate cancer [14]. These findings indicated that VISTA probably represented another compensatory inhibitory pathway, after the cancer’s resistance to anti-PD-L1/ (cytotoxic lymphocyte antigen 4) CTLA4 therapy; a combination of VISTA and PD-1/CTLA4 blockade might be a promising new option for cancer treatment. This study investigated the expression of VISTA in HCC tumors and analyzed its association with clinicopathological features, TILs in the tumor microenvironment, and clinical outcomes, which provide the basis for further VISTA blockade immunotherapies in patients with HCC.

## Methods

### Patients, cohorts, and tissue microarrays

Two HCC tissue microarray (HCC-TMA) chips containing a total of 183 pairs of HCC and matched adjacent tissues were obtained from Shanghai Biochip Company Ltd. Samples for TMA were collected using 1.5-mm diameter core needles from a spot of tumors with the most representative histology of each surgical specimen. All patients were followed up for at least 4 years, with the median follow-up period being 43 months (range: 1–80 months). For the use of these clinical materials for research purposes, prior patient consent and approval from the Institute Research Ethics Committee were obtained.

The Cancer Genome Atlas (TCGA) data were retrieved from the online data repository http://www.cbioportal.org/data_sets.jsp. A total of 372 patients were included in the TCGA cohort with mRNA expression profiling, clinical features, and follow-up information. The median follow-up period was 19.3 months (range: 0.03–120.7 months).

The key variables of these two cohorts, including demographic and clinical information, are provided in Additional file 1: Table S1.

### Immunohistochemistry

IHC staining was performed using a Dako Envision System (Dako, Carpinteria, CA, USA) following the manufacturer’s protocol. Tumor sections were assessed immunohistochemically using anti-VISTA (dilution 1:500, clone D1L2G, Cell Signaling, Danvers, United States of America) and anti-CD8 (dilution 1:60, clone: C8/144B, Gene Tech (Shanghai) Co. Ltd) solutions. Serial sections from the HCC-TMA were used for analyzing VISTA and CD8. The IHC-stained tissue sections were scored separately by two pathologists (XYL and FLY) blinded to the clinical parameters.

### Evaluation of immunostaining

VISTA expression was evaluated on the basis of tumor cells (TCs) and tumor-infiltrating immune cells (ICs). For TCs, the proportion of VISTA-positive cells was estimated as the percentage of the total TCs stained at any intensity. TCs typically showed membranous staining with a variably strong component of cytoplasmic staining. For tumor-infiltrating ICs, the percentage of VISTA-positive tumor-infiltrating ICs, which included macrophages, dendritic cells, and lymphocytes, occupying the tumor was recorded. VISTA-positive tumor-infiltrating ICs were typically seen as variably sized aggregates towards the periphery of the tumor mass, in stromal bands dissecting the tumor mass, as single cells scattered within the stroma, or within tumor-infiltrating IC aggregates. For the purpose of statistical evaluation, we defined a final VISTA staining score of ≥5% (TC or IC) as the cutoff value, which referred to the PD-L1 IHC evaluation; an SP142 clone was used for this process [15]. The percentage of CD8+ lymphocytes compared with that of the nucleated cells in the stromal compartments was assessed. A staining score of ≥10% for each core was set as the parameter for high density of CD8+ lymphocytes, according to the degree of cell densities.

### mRNA expression profiling analysis

For HCCs included in the TCGA cohort, the results shown in this study are based upon data generated by TCGA Research Network at: http://cancergenome.nih.gov/. Normalized RNA-Seq by Expectation Maximization (RSEM) files were downloaded from TCGA for 372 HCC patients. Experimental procedures regarding RNA extraction, mRNA library preparation, quality control, and subsequent data processing for the quantification of gene expression have been previously reported [16]. The gene expression cutoff value was chosen as the median for the entire dataset.

### Statistical analysis

Statistical analyses were performed using GraphPad Prism (version 7.01), and SPSS (version 22.0) (SPSS, Inc.). Chi-square tests were used to analyze the correlation between VISTA protein expression and clinical pathological variables. The correlation between the expression of VISTA and CD8 + TILs was analyzed by the Spearman’s rank correlation test. The survival curves were estimated by the Kaplan-Meier method. The Cox regression models were used to investigate the relationships between correlative factors and HCC overall survival (OS). All statistics were 2-sided, and the statistical significance was defined as *p* < 0.05.

## Results

### VISTA expression and clinicopathological characteristics

**Fig. 1:**
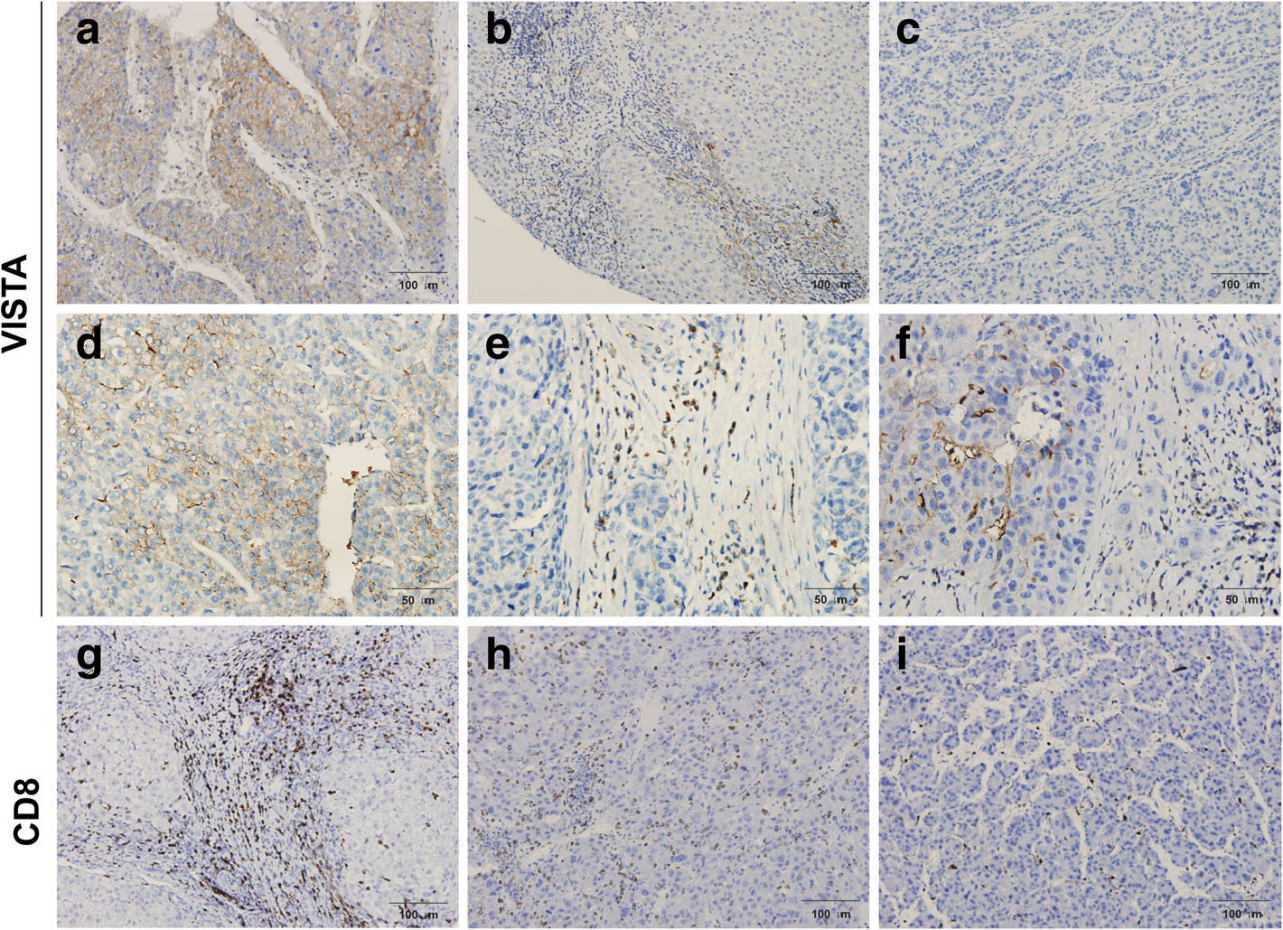
Immunohistochemical staining of VISTA and CD8 in patients with HCC. Representative staining intensity (**a**, strong; **b**, moderate; and **c**, negative) of VISTA protein was detected in HCC tissues by immunohistochemistry. Positive membranous VISTA staining was shown in tumor cells (**d**), immune cells (**e**), and both types (**f**). Representative staining density (**g**, strong; **h**, moderate; and **i**, negative) of CD8-positive tumor infiltrating lymphocytes was detected in HCC tissues by immunohistochemistry. HCC, hepatocellular carcinoma; VISTA, V-domain Ig suppressor of T cell activation

Of the 183 HCC cases (157 males and 26 females with the median age of 53), VISTA expression was detected in 29.5% (54/183) of HCC tissues. Representative IHC staining intensity of VISTA was shown in Fig. 1a-c and Additional file 2: Figure S1. Notably, VISTA protein can be detected in TCs and/or ICs of HCC tissues (Fig. 1d-f). In total, 16.4% (30/183) tissues showed VISTA expression in TCs, 16.9% (31/183) tissues showed VISTA expression in ICs, and 3.8% (7/183) tissues showed VISTA expression in both TCs and ICs. CD8-positive TILs were also detected in the patients whose serial sections were analyzed. Representative IHC staining density of CD8 was shown in Fig. 1g-i and Additional file 3: Figure S2. A high density of CD8 + TILs was showed in 33.3% (61/183) HCC tissues.

VISTA expression according to patient characteristics and tumor pathological features are presented in Table 1. VISTA expression was significantly associated with cases with a high pathological grading (III-IV, *p* = 0.038) and without liver cirrhosis (*p* = 0.011). Meanwhile, VISTA expression showed a significant correlation with high density of CD8 + TILs (*p* < 0.001). However, there were no correlations between VISTA expression and gender, age, tumor size, or TNM stage. We further analyzed the correlations between VISTA-positive cell types (TCs/ICs) and their clinicopathological characteristics. The results showed that both VISTA staining in TCs and ICs were significantly associated with a high density of CD8 + TILs (TC, *p* < 0.001; IC, *p* = 0.001). However, VISTA staining showed a negative correlation with liver cirrhosis (*p* = 0.019) and tumor size (*p* = 0.042) only in TCs, and none of the VISTA-positive cell types showed a correlation with pathological grading.

**Table 1:** Clinicopathological correlation of VISTA expression in 183 HCC patients

Feature	Total (N)	VISTA		*P*	VISTA (tumor cell)		*P*	VISTA (immune cell)		*P*
		Pos (n, %)	Neg (n, %)		Pos (n, %)	Neg (n, %)		Pos (n, %)	Neg (n, %)	
Age(years)										
> 50	108	36(33.3%)	72(66.7%)	0.173	20(18.5%)	68(81.5%)	0.351	22(20.4%)	86(79.6%)	0.138
≤ 50	75	18(24.0%)	57(76.0%)		10(13.3%)	65(86.7%)		9(12.0%)	66(88.0%)	
Gender										
Male	157	48(30.6%)	109(69.4%)	0.438	27(17.2%)	130(82.8%)	0.470	28(17.8%)	129(82.2%)	0.428
Female	26	6(23.1%)	20(76.9%)		3(11.5%)	23(88.5%)		3(11.5%)	23(88.5%)	
Tumor size										
≤ 5 cm	91	31(34.1%)	60(65.9%)	0.804	20(22.0%)	71(78.0%)	0.042^*^	19(20.9%)	72(79.1%)	0.158
> 5 cm	92	23(25.0%)	69(75.0%)		10(10.9%)	82(89.1%)		12(13.0%)	80(87.0%)	
TNM stage										
I	73	25(34.2%)	48(65.8%)	0.294	13(17.8%)	60(82.8%)	0.272	17(23.3%)	56(76.7%)	0.261
II	60	14(23.3%)	46(76.7%)		7(11.7%)	53(88.3%)		8(13.3%)	52(86.7%)	
III-IV	42	15(35.7%)	27(64.3%)		10(23.8%)	32(76.2%)		6(14.3%)	36(85.7%)	
Pathological grade										
I-II	119	29(24.4%)	90(75.6%)	0.038^*^	16(13.4%)	103(86.6%)	0.142	18(15.1%)	101(84.9%)	0.372
III-IV	64	25(39.1%)	39(60.9%)		14(21.9%)	50(78.1%)		13(20.3%)	51(79.7%)	
Liver cirrhosis										
Yes	69	26(22.8%)	88(77.2%)	0.011^*^	13(11.4%)	101(88.6%)	0.019^*^	17(14.9%)	97(85.1%)	0.347
No	114	28(40.6%)	41(59.4%)		17(24.6%)	52(75.4%)		14(20.3%)	55(79.7%)	
CD8^+^TILs										
Yes	61	31(50.8%)	30(49.2%)	0.000^*^	19(31.1%)	42(68.9%)	0.000^*^	18(29.5%)	43(70.5%)	0.001^*^
No	122	23(18.9%)	99(81.1%)		11(9.0%)	111(91.0%)		13(10.7%)	109(89.3%)	

*VISTA* V-domain Ig suppressor of T cell activation, *Pos* positive, *Neg* negative, *HCC* hepatocellular carcinoma, *TILs* tumor-infiltrating lymphocytes

^*^The values had statistically significant differences

### Comparison of prognostic impacts of VISTA expression in tumor cells and immune cells

**Fig. 2:**
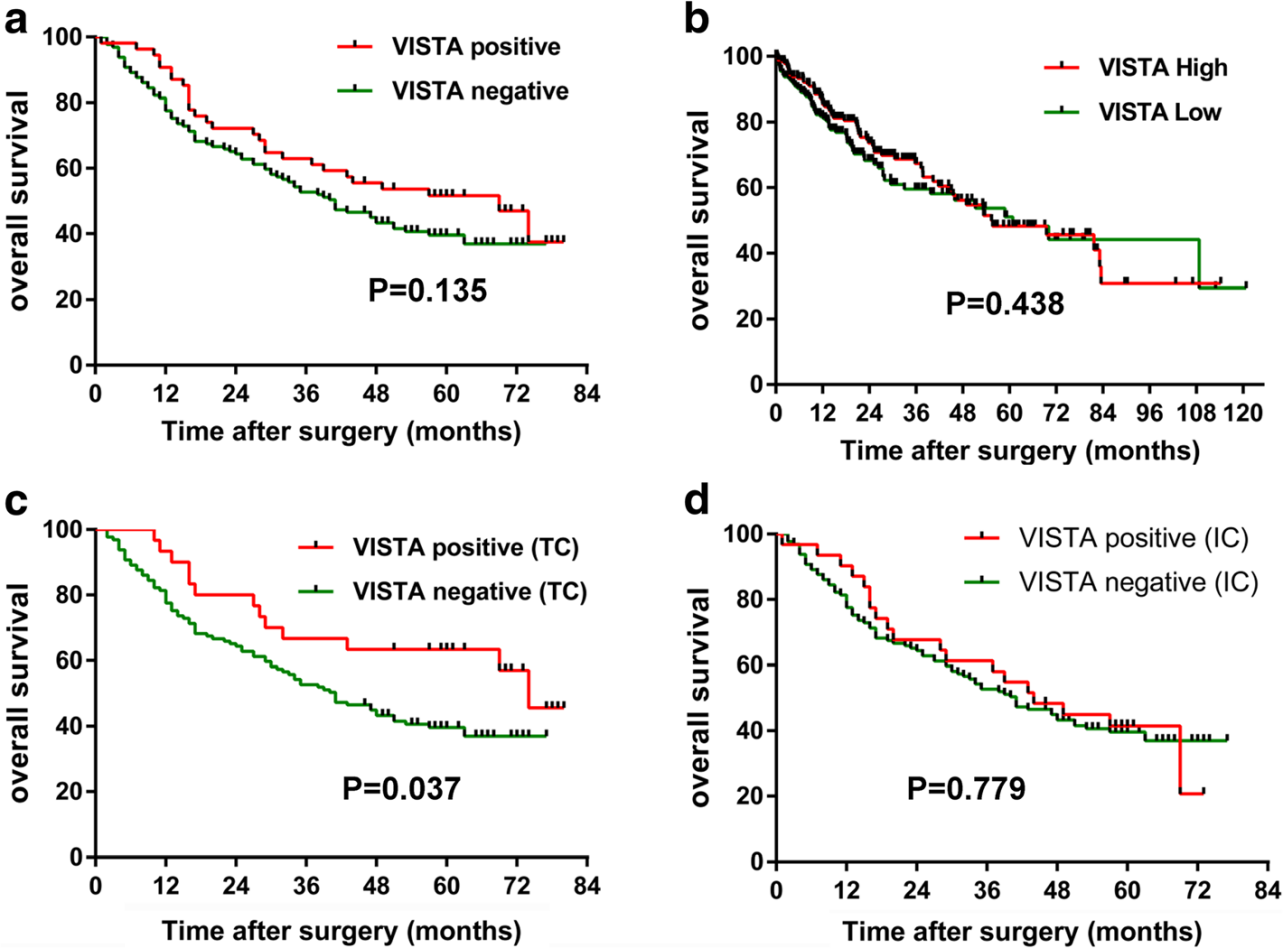
Kaplan-Meier curves showing overall survival (OS) of patients with hepatocellular carcinoma (HCC) based on VISTA status. Kaplan-Meier curves of OS in HCC tissue microarray (TMA) cohort (**a**) and The Cancer Genome Atlas (TCGA) cohort (**b**) according to VISTA protein and mRNA expression. Kaplan-Meier curves of OS in the HCC-TMA cohort classified on the basis of whether they are positive in tumor cells (**c**) or in immune cells (**d**). TC, tumor cell; IC immune cell. VISTA, V-domain Ig suppressor of T cell activation

Among all HCC cases with a follow-up of at least 4 years, patients with a high VISTA expression showed no significant difference in OS compared to those with low VISTA expression in the Kaplan–Meier curves analysis (69 months vs 41 months, *p* = 0.135) (Fig. 2a). To further determine whether VISTA expression was correlated with the prognoses of patients with HCC, we included a HCC-TCGA cohort to analyze *VISTA* mRNA expression and the OS of patients with HCC. The gene expression cutoff value was chosen as the median for the entire dataset. The results also failed to show any significant difference in OS between VISTA-high and -low (55.4 months vs 60.8 months, *p* = 0.438) patients (Fig. 2b).

We had discovered the difference of VISTA expression in TCs and ICs. Next, we sought to explore whether VISTA-positive cell types affect the prognosis of patients with HCC. As the result showed, it was the patients with VISTA-positive staining in TCs (74 months vs 41 months, *p* = 0.037), but not in ICs (44 months vs 41 months, *p* = 0.779), that showed significantly prolonged OS compared to those with negative-VISTA expression (Fig. 2c and d), which suggested a favorable survival of patients with HCC with VISTA staining in TCs.

Furthermore, we performed the univariate and multivariate analysis of OS in patients with HCC. Univariate analysis for OS indicated that TNM stage (*p* < 0.001), tumor size (*p* = 0.002), VISTA expression in TC (*p* = 0.038), and CD8 + TILs (*p* = 0.008) were significantly correlated with OS. Multivariate Cox regression analysis showed that TNM stage (*p* = 0.001) and CD8 + TILs (*p* = 0.024) were two independent prognostic factors for OS, while VISTA expression and tumor size were not (Table 2).

**Table 2:** Univariate and multivariate analyses of prognostic parameters in 183 HCC patients by Cox-regression analysis

Variables	Univariate		Multivariate	
	*P*	HR (95% CI)	*P*	HR (95% CI)
Gender				
Male versus Female	0.146	1.562 (0.856–2.851)		
Age(year)				
> 50 versus ≤50	0.787	1.055 (0.714–1.561)		
Tumor size (cm)				
≤ 5 versus > 5	0.002^*^	1.847 (1.252–2.724)	0.218	1.314 (0.851–2.028)
TNM stage				
I versus II versus III-IV	0.000^*^	1.617 (1.258–2.077)	0.001^*^	1.627 (1.235–2.143)
Pathological grade				
I-II versus III-IV	0.748	1.068 (0.717–1.590)		
Liver cirrhosis				
Yes versus No	0.460	0.862 (0.582–1.277)		
VISTA				
Positive versus Negative	0.139	0.720 (0.466–1.113)		
VISTA (tumor cell)				
Positive versus Negative	0.038^*^	0.536 (0.297–0.966)	0.128	0.610 (0.323–1.152)
VISTA (immune cell)				
Positive versus Negative	0.890	1.036 (0.630–1.703)		
CD8^+^TILs				
Positive versus Negative	0.008^*^	0.556 (0.361–0.858)	0.024^*^	0.586 (0.368–0.932)

*VISTA* V-domain Ig suppressor of T cell activation, *HCC* hepatocellular carcinoma, *TILs* tumor-infiltrating lymphocytes, *HR* hazard radio, *CI* confidence internal

^*^The values had statistically significant differences

### Characterization of the immune microenvironment based on VISTA and CD8 + TILs

**Fig. 3:**
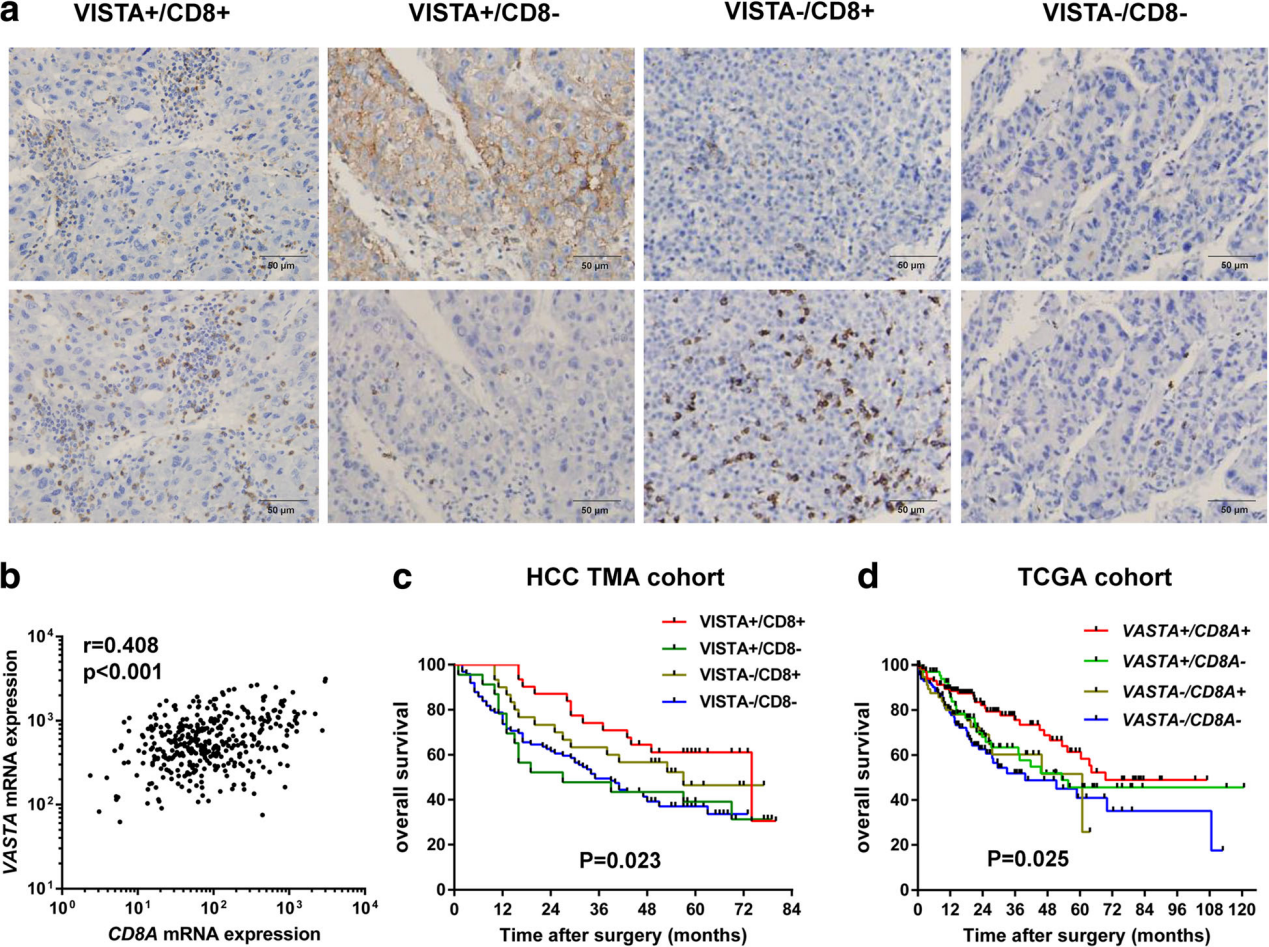
Classification of HCC immune microenvironments based on VISTA and CD8 + TILs. **a** Four immune subtypes based on VISTA and CD8 immunohistochemical staining. **b** Correlation of *VISTA* and *CD8A* mRNA analyzed from the TCGA database. Kaplan-Meier curves of OS in the HCC-TMA cohort **c** and the TCGA cohort **d** according to four immune subtypes based on VISTA and CD8 expression. HCC, hepatocellular carcinoma; VISTA, V-domain Ig suppressor of T cell activation; TCGA, The Cancer Genome Atlas; TMA, tissue microarray; OS, overall survival

Based on the TCGA mRNA expression profile analysis, we further confirmed the positive correlation between *VISTA* and *CD8A* mRNA expression (*r* = 0.408, *p* < 0.0001, Fig. 3b). Given that VISTA IHC expression was correlated with the density of CD8 + TILs, we sought to determine the HCC immune microenvironment-based VISTA and CD8 + TILs. From the 183 HCC serial sections for VISTA and CD8 + TILs IHC analysis, we classified 4 immune subtypes: VISTA+/CD8+ (16.9%, 31/183), VISTA+/CD8- (12.6%, 23/183), VISTA-/CD8+ (16.4%, 30/183), and VISTA-/CD8+ (54.1%, 99/183) (Fig. 3a). We subsequently assessed the relationship between the 4 immune subtypes and the prognosis of patients with HCC. We performed the Kaplan–Meier curves analysis in two independent cohorts: the HCC TMA cohort and the TCGA cohort. The HCC TMA cohort indicated that those with dual-positive VISTA and CD8 showed a significantly longer OS than other subsets (VISTA+/CD8+ vs VISTA+/CD8- vs VISTA-/CD8+ vs VISTA-/CD8-: 74 vs 27 vs 57 vs 35 months, *p* = 0.023) subtypes (Fig. 3c). Consistent with the HCC TMA cohort, the TCGA cohort also showed a predominant OS benefit in the *VISTA+/CD8A+* subtype. (*VISTA+/CD8A+* vs *VISTA*+/*CD8A*- vs *VISTA*-/*CD8A*+ vs *VISTA-/CD8A-*: not reached vs 53.3 vs 60.8 vs 39.7 months, *p* = 0.025) (Fig. 3d).

## Discussion

To our knowledge, this is the first study to analyze VISTA protein expression in HCC. We discovered VISTA protein expression in HCC TCs and tumor infiltrating ICs. Previous study on mouse models and cell lines suggest that VISTA is exclusively expressed on leukocytes infiltrating the tumor [17]. Recent study that explored the expression of VISTA in gastric carcinoma had first identified VISTA expressed in TCs using a distinct cytoplasmic staining. However, they had observed that VISTA expression in TCs was only found in a small subset of gastric carcinoma cells compared to its predominant expression in ICs (8.8% vs 83.6%) [18]. Our study demonstrated that the VISTA protein was equally expressed in HCC TCs (16.4%) and ICs (16.9%), and displayed different prognoses in OS. This discrepancy is likely due to biological differences between mouse models, cell lines, or human patients, tumor types, and the immunohistochemical evaluation for VISTA expression. Although the exact VISTA-binding partners are not yet known, several studies have demonstrated that VISTA serves as both a ligand for antigen presenting cells, and a receptor for T cells, and that VISTA suppresses T cell activation [10, 19, 20]. What is the exact role of VISTA expressed on tumor cells is still unknown, further study need to determine the significances and functions of VISTA expressed on different cell types including tumor cell, antigen presenting cells, and T cells. These explorations can be of great help to understand the potential value of VISTA for immunotherapy.

VISTA has recently been identified as a negative checkpoint regulator, and a potent suppressor of T-cell proliferation and activation [10, 21], which seems to produce a poor prognosis in theory. However, we have demonstrated VISTA expression in TCs resulted in a favorable prognosis. This finding indicated that there may be different functions and mechanisms involved when VISTA is expressed in TCs and ICs. We found VISTA expressed in TCs was reversely correlated with tumor size (*p* = 0.042) and liver cirrhosis (*p* = 0.019), which supported VISTA expressed in TCs may act as a tumor-suppressor gene that inhibits tumor cell proliferation and progression. Furthermore, we have found that VISTA expression was significantly correlated with the density of CD8 + TILs, which implied that VISTA may affect potential signaling in the tumor microenvironment, to recruit T-cell infiltration and subsequently attack the TCs. Besides, recent published study of VISTA in non-small cell lung cancer also supported our results that elevated expression of VISTA measured exclusively in the tumor area, was significantly associated with longer 5-year overall survival [22].

Recent study have demonstrated negative immune checkpoint regulation by VISTA, which represented an important potential mechanism of acquired resistance in melanoma patients treated with anti-PD-1 [12]. Similarly, Gao et al. [14] also identified that VISTA represented another compensatory inhibitory pathway in prostate tumors after ipilimumab therapy. These studies highlighted a possibility that increased VISTA expression can be a potential mechanism for anti-PD-1 and anti-CTLA4-acquired resistance. Meanwhile, Liu et al. [13] found the nonredundant role of VISTA, which was distinct from the PD-1/PD-L1 pathway in controlling T cell activation. Immunofluorescent staining displayed that VISTA and PD-1 are located in different T lymphocytes. We also analyzed the correlation between PD-L1 or PD-1 and VISTA mRNA expression based on TCGA analysis; the results showed a weak correlation between PD-L1 and VISTA (*r* = 0.157), as well as PD-1 and VISTA (*r* = 0.228) (data not shown). These results further supported the possibility that a combination of VISTA and PD-1/CTLA4 blockade might be a promising option to overcome checkpoint inhibitor resistance. However, before the combination treatment, we should first identify the different roles and mechanisms of primary and acquired expressions of VISTA, in order to design a reasonable treatment strategy.

It has been proposed that four different types of tumor immune microenvironment exist based on the presence or absence of TILs and PD-L1 expression [5, 6, 23]. VISTA shared homology with PD-L1 and displayed a similar cell staining pattern. In this study, we classified HCC immune microenvironments based on VISTA and CD8 + TILs. We had identified different prognoses among the 4 immune subtypes. Patients with positive VISTA and CD8 expression showed a significantly longer OS than either VISTA- or CD8-positive, or both VISTA- and CD8-negative patients. Previous studies demonstrated PD-L1 positive with presence of TILs mostly induce by interferon-gamma (IFN-γ) mediated pathways that confer adaptive immune resistance [24, 25]. Whether VISTA can be induced by IFN-γ or other cytokines released from infiltrating T lymphocytes was unknown. Further study should clarify the genomic and immune profiles of HCC TMEs based on VISTA and CD8 + TILs.

## Conclusions

We showed that VISTA protein expression in HCC tissues displayed cell-specific and prognostic diversity. VISTA expression was significantly associated with CD8 + TILs. HCC immune microenvironment based on VISTA and CD8 + TILs are promising. VISTA+/CD8+ patients showed a favorable TME and better OS. This work established the specific significance of VISTA expression in the prognosis and immune microenvironment of HCC. Further studies should explore the mechanisms and functions of VISTA in the setting of anti-tumor immunity for patients with HCC.

## Additional files

Additional file 1: **Table S1.** Demographic and clinicopathologic characteristics of hepatocellular carcinoma samples in 2 independent cohorts. (DOCX 19 kb)

Additional file 2: **Figure S1.** Immunohistochemically staining of PD-L1 proteins in patients with hepatocellular carcinoma. Representative images of different immunostaining score of PD-L1 protein in tumor cells (TCs) and tumor-infiltrating immune cells (ICs). (TIF 72870 kb)

Additional file 3: **Figure S2.** Immunohistochemically staining of CD8 proteins in patients with hepatocellular carcinoma. Representative images of different density of CD8+ tumor infiltrating lymphocytes. (TIF 56816 kb)

## Abbreviations

CTLA4: Cytotoxic lymphocyte antigen 4
HCC: Hepatocellular carcinoma
ICs: Immune cells
IFN-γ: Interferon-gamma
IHC: Immunohistochemistry
OS: Overall survival
PD-1: Programmed death 1
PD-L1: Programmed cell death 1 ligand 1
RSEM: RNA-Seq by Expectation Maximization
TCGA: The Cancer Genome Atlas
TCs: Tumor cells
TILs: Tumor-infiltrating lymphocytes
TMAs: Tissue microarrays
TMEs: Tumor microenvironments
VISTA: V-domain Ig suppressor of T cell activation
