# Supplementary material for: VISTA expression associated with CD8 confers a favorable immune microenvironment and better overall survival in hepatocellular carcinoma
Source: BMC Cancer. 2018 May 2;18:511. doi: 10.1186/s12885-018-4435-1 (PMC5932869; doi:10.1186/s12885-018-4435-1)
Supplement: Supplementary file 1 — Table S1. Demographic and clinicopathologic characteristics of hepatocellular carcinoma samples in 2 independent cohorts. (DOCX 19 kb) [file 12885_2018_4435_MOESM1_ESM.docx]

**Table S1**. Demographic and clinicopathologic characteristics of hepatocellular carcinoma samples in 2 independent cohorts.

|  | **HCC TMA Cohort** | **TCGA Cohort** |
| --- | --- | --- |
| **No. of patients** | 183 | 372 |
| **Age** |  |  |
| **>50** | 108 (59.0) | 294 (79.0) |
| **≤50** | 75 (41.0) | 78 (21.0) |
| **Gender** | |  |
| **Male** | 157 (85.8) | 251 (67.5) |
| **Female** | 26 (14.2) | 121 (32.5) |
| **Tumor size** |  |  |
| **≤5 cm** | 91 (49.7) | 182 (49.2) |
| **>5 cm** | 92 (50.3) | 190 (50.8) |
| **Stage** |  |  |
| **I** | 73 (39.9) | 172 (46.2) |
| **II** | 60 (32.8) | 86 (23.1) |
| **III-IV** | 42 (22.9) | 91 (24.5) |
| **NA** | 8 (4.4) | 23 (6.2) |
| **Pathological grade** | |  |
| **I-II** | 119 (65.0) | - |
| **III-IV** | 64 (35.0) | - |
| **Liver cirrhosis** | |  |
| **Yes** | 69 (37.7) | 79 (21.3) |
| **No** | 114 (62.3) | 134 (36.0) |
| **NA** | 0 | 159 (42.7) |
| **Vital status** | |  |
| **Living** | 77 (42.1) | 242 (66.1) |
| **Deceased** | 106 (57.9) | 130 (35.5) |

Abbreviations: HCC, hepatocellular carcinoma; TMA, tissue microarray; TCGA, The Cancer Genome Atlas; NA, not available.
