# Supplementary figures and images for: VISTA expression associated with CD8 confers a favorable immune microenvironment and better overall survival in hepatocellular carcinoma
Source: BMC Cancer. 2018 May 2;18:511. doi: 10.1186/s12885-018-4435-1 (PMC5932869; doi:10.1186/s12885-018-4435-1)

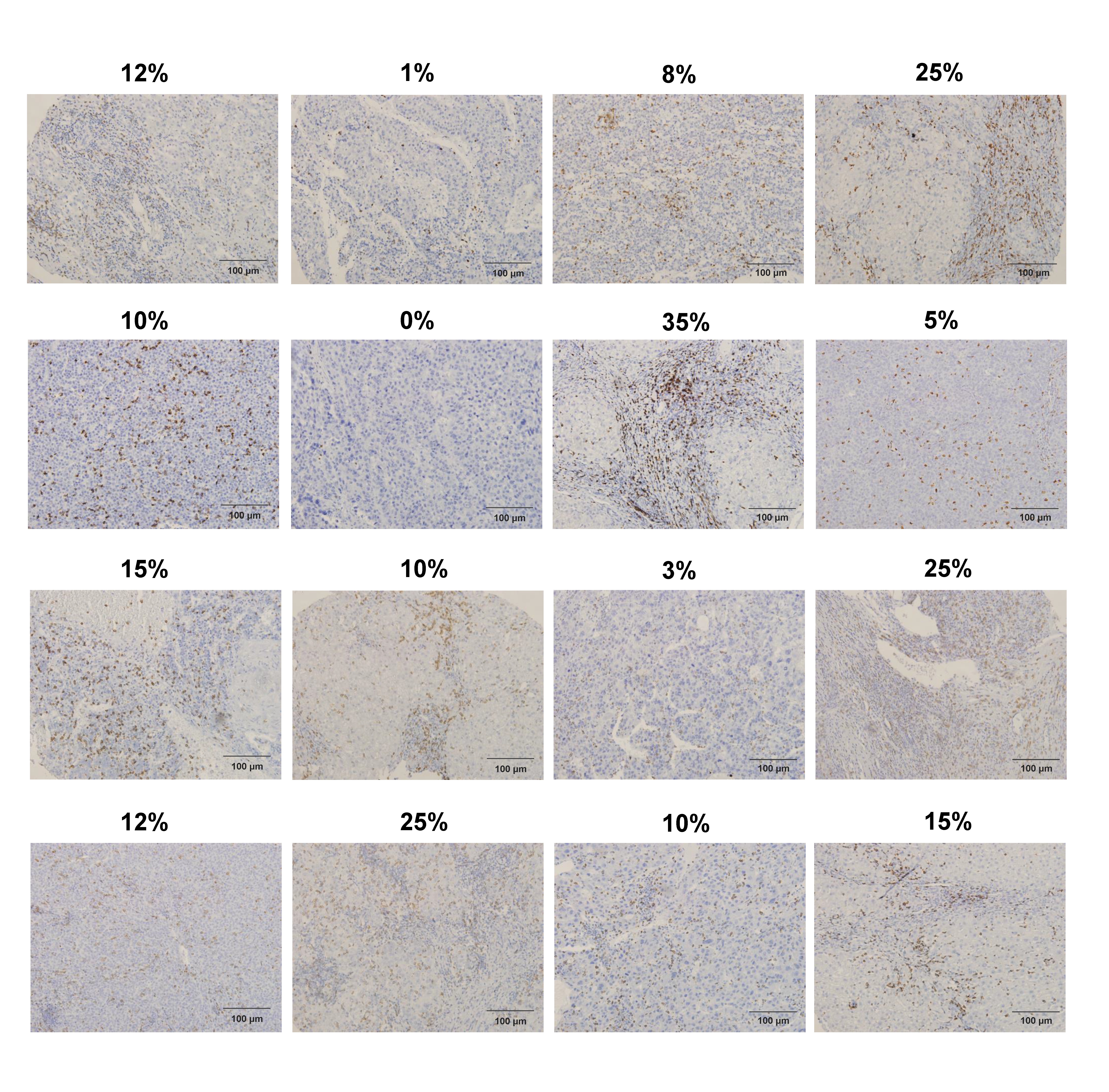

Supplement: Supplementary file 3 — Figure S2. Immunohistochemically staining of CD8 proteins in patients with hepatocellular carcinoma. Representative images of different density of CD8+ tumor infiltrating lymphocytes. (TIF 56816 kb) [file 12885_2018_4435_MOESM3_ESM.tif]
